# Analysis of the relation between adverse events and overall survival in patients treated with pembrolizumab as a first-line treatment for metastatic NSCLC

**DOI:** 10.1186/s40360-023-00663-0

**Published:** 2023-05-15

**Authors:** Lisa Faoro, Adriana Brusegan, Alberto Russi, Vincenzo Calderone, Alma Martelli, Ettore Marranconi, Debora Carpanese, Elena Berti, Marina Coppola

**Affiliations:** 1 Department of Pharmacy, Specialization School in Hospital Pharmacy, University of Pisa, Pisa, Italy; 2 Pharmacy Unit, Veneto Institute of Oncology IOV-IRCCS, Padua, Italy; 3 Immunology and Molecular Oncology Unit, Veneto Institute of Oncology IOV-IRCCS, Padua, Italy

**Keywords:** First-line monotherapy, Non–small cell lung cancer, PD-L1 tumour proportion score, Pembrolizumab, Toxicity

## Abstract

**Background:**

Many trials supported pembrolizumab as a first-line monotherapy to significantly improve overall survival (OS) in selected patients with previously untreated metastatic Non–Small Cell Lung Cancer (mNSCLC) and a PD-L1 TPS of ≥50% without EGFR/ALK mutations. The aim of this study was to reveal the correlation between OS and adverse events in real-world settings after 42 months.

**Methods:**

This retrospective observational study involved 98 patients with mNSCLC, TPS ≥ 50%, and no EGFR/ALK aberrations. Patients were treated with pembrolizumab (200 mg q3w) as a first-line treatment. Clinical data, including PD-L1 expression, Performance Status (ECOG-PS), treatment duration, toxicity, and outcomes were retrieved from local electronic medical records and from the Italian Regulatory Agency Registry.

**Results:**

The cohort’s main characteristics were as follows: median age 73 [44-89] years, 64.3% were male and 35.7% were female, an ECOG-PS score of 0 (*n* = 73) and 1 or 2 (*n* = 25), and a PD-L1 > 90% in 29.6% of patients. The entire cohort had stage IV NSCLC at diagnosis. The median number of cycles was 8.5 at a median follow-up of 13 months. The median OS of 13.6 months (95% CI: 11.7-NA) was not influenced by sex and PD-L1, but was significantly associated with ECOG-PS (*p* = 0.02). Immune-Related Adverse Events (irAEs) occurred in 77.5% of patients (30.1% cutaneous, 27.5% gastrointestinal, and 20.4% endocrinological), but no grade 4 or 5 irAEs were identified. Patients experiencing any type of toxicity had a significantly longer median OS (20.39 months, 95% CI: 13.08-NA) than those with no toxicities (6.46 months, 95% CI: 1.41-NA, *p* = 0.006).

**Conclusion:**

The percentage of irAEs detected was comparable to that reported in KEYNOTE-024 and KEYNOTE-042. These real-world findings demonstrated the significant correlation between OS and cutaneous toxicities.

## Key points

This study was designed to verify the effectiveness of toxicities associated with pembrolizumab in patients with mNSCLC in a real-world setting.

Patients with any kind of toxicity showed a significantly higher median OS than those with no toxicities.

The calculated percentage of irAEs (77.5%) proved to be comparable with the percentages reported in KEYNOTE-024 and KEYNOTE-042 (76.6 and 63%, respectively).

## Background

According to the World Health Organization (WHO), non–small cell lung cancer (NSCLC) is a common and deadly malignancy with over 2.21 million new diagnoses and over 1.8 million associated deaths per year worldwide. In particular, NSCLC is the most common type of lung cancer, accounting for 84% of all lung cancer diagnoses, as reported by international cancer statistics (https://www.cancer.net/cancer-types/lung-cancer-non-small-cell/statistics).

In the last decade, immune checkpoint inhibitors (ICIs) targeting programmed death-1 (PD-1) or programmed death-ligand 1 (PD-L1) have proved to be a promising treatment option for patients with advanced/metastatic NSCLC. However, a substantial proportion of patients will not benefit from these treatments, and robust biomarkers are required to help clinicians select patients who are most likely to benefit [1]. In fact, pembrolizumab interferes with the adaptive immune system activation on the PD-1 checkpoint mostly located on the surface of the lymphocytes type T. The triggers of inflammatory and immunological reactions are correlated to the mechanism of action and can be exacerbated by genetic predisposition, environmental relation and presence of target antigens in both tumour and involved tissue.

The therapeutic blockade of PD-1, expressed on the T cells by the linkage of PD-L1 and PD-L2 expressed by the tumour cells, prevents the downregulation of the T cell effector function, allowing the lymphocytes to mediate tumour cell death. Human and humanized monoclonal antibodies that inhibit the PD-1 receptor, such as nivolumab and pembrolizumab, showed a more favourable tolerance profile and improved results when compared to chemotherapy [2, 3].

Data from the phase 1 KEYNOTE-001 and phase 3 KEYNOTE-010 studies [4, 5] indicated that patients with advanced NSCLC and a PD-L1 tumour proportion score (TPS) of 50% or greater are more likely to respond to pembrolizumab than those with a lower TPS. Based on these results, the TPS percentage was identified as a discriminating parameter in this work.

The Eastern Cooperative Oncology Group Performance Status (ECOG-PS) is an international scale that standardizes criteria for measuring how cancer impacts the patient’s daily living abilities. It describes a patients’ level of functioning in terms of self-care, daily activity, and physical ability. The ECOG scale ranges between 0 (fully active) and 5 (dead). In addition to TPS %, the ECOG-PS has been identified as an additional discriminating parameter because this value reflects the overall clinical status of the patients, indirectly reflecting the immune system’s ability to respond.

Current clinical decisions for advanced NSCLC in first-line treatment are based on the presence of genetic aberrations, such as sensitizing epidermal growth factor receptor (EGFR) mutations, and translocations of anaplastic lymphoma kinase (ALK). As a result, the KEYNOTE-001 trial enrolled untreated patients affected by NSCLC, treated them with pembrolizumab at a dose of 10 mg per kilogram every 2 or 3 weeks, and demonstrated that 12-month Overall Survival (OS) was higher among patients with a PD-L1 TPS ≥ 50% (85%) when compared with the overall population (71%) [6].

The international, randomized, open-label, phase 3 KEYNOTE-024 trial compared pembrolizumab (administered at a fixed dose of 200 mg every 3 weeks) [7] to the investigator’s choice of cytotoxic chemotherapy as first-line therapy in patients with advanced NSCLC and a PD-L1 TPS of 50% or greater. Based on this trial’s results, pembrolizumab was approved as a first-line treatment for metastatic NSCLC with a PD-L1 TPS ≥ 50% [8]. The KEYNOTE-024 trial demonstrated a median OS of 30.0 months (95% CI: 18.3-NA) in the pembrolizumab arm versus 14.2 months (95% CI: 9.8-19.0) in the chemotherapy group, with a Hazard Ratio (HR) of 0.63 (95% CI: 0.47-0.86; *p* = 0.002). Another randomized trial, KEYNOTE-042, evaluated pembrolizumab in NSCLC first-line treatment with a PD-L1 score ≥ 1%. The subgroup of patients with a PD-L1 ≥ 50% showed a HR for OS of 0.69 (95% CI: 0.56-0.85; *p* = 0.0003), confirming the results of the previously cited trial. The toxicity profile revealed that grade 3 or higher adverse reactions occurred in 63% of patients, whereas endocrinological adverse reactions (hypothyroidism) manifested in 12% of patients in the pembrolizumab group [9].

The results of KEYNOTE-024 changed the landscape for advanced NSCLC patients with a TPS ≥ 50%: pembrolizumab in monotherapy became the standard of care in first-line treatment. However, results from recent phase 3 trials evaluating combinations of immunotherapy and chemotherapy may probably further change the therapeutic strategy. Among these, the KEYNOTE-189 trial [10] compared the combination of pembrolizumab or placebo plus pemetrexed-platinum in the first-line treatment of patients with no squamous advanced NSCLC. After a median follow-up of 23.1 months, the estimated rate of OS was 22.0 months (95% CI: 19.5-25.2) in the pembrolizumab plus pemetrexed and platinum group versus 10.7 (95% CI: 8.7-13.6) months in the placebo plus pemetrexed and platinum group.

This paper discusses available evidence regarding the need to consider further clinical characteristics when selecting patients with metastatic NSCLC as potential candidates for single-agent anti-PD-1/PD-L1 therapy. Recommendations on the use of ICIs in clinically challenging populations are also provided.

In order to correctly analyse this treatment’s safety profile, it is important to underline the mechanisms of autoimmunity linked to antigens that are localized in both tumour and healthy tissues. Overall immune system involvement may exacerbate different types of immune related Adverse Events (irAEs). The discussion section will address these events and the influence of correlated toxicities. The use of corticosteroids is precluded at the start of immunotherapy because of the suspected negative influence on the mechanisms of action of ICIs. However, recent findings reveal that the use of concomitant steroids for non-cancer-related symptoms has no impact on OS analysis. This topic has been subject of specific debate [11].

In order to address the dearth of references regarding the role that those adverse reactions play in survival, this analysis investigates the significance of irAEs on the outcomes. Thus, the purpose of this study was to investigate the relationship between OS and toxicities in patients treated with pembrolizumab in the real-world, outside of restrictive clinical trial settings.

## Methods

### Objectives

This research aims to confirm the effectiveness and toxicities of pembrolizumab (administered at 200 mg every 3 weeks) in the treatment of NSCLC patients in a real-world setting. The results were compared and discussed in relation to previous randomized clinical trials (RCT) and real-world studies. The study also investigated whether the occurrence of different types of immunological toxicities can affect OS.

### Study design and patients

This retrospective observational study was conducted at the Veneto Institute of Oncology (IOV) IRCCS, Northern Italy. All eligible patients exhibited metastatic NSCLC with a PD-L1 ≥ 50%, and a PS of 0, 1, or 2 without evidence of renal or hepatic dysfunctions. Patients with EGFR or ALK aberrations were excluded from the study as per the AIFA (Italian Regulatory Agency) Registry’s eligibility criteria.

The enrolment period started in July 2017 and ended in February 2020, and 98 patients with NSCLC were treated with pembrolizumab as first-line therapy. All patients were followed from the first treatment’s administration up to December 2020.

### Data collection

Clinical data, including PD-L1 expression, Performance Status (ECOG-PS), treatment duration, toxicity (using Common Terminology Criteria for Adverse Events, CTCAE v.5.0), and outcomes were obtained from electronic medical records of Veneto Institute of Oncology (IOV) IRCCS. Anonymized patient data were also retrieved from the AIFA Registry. The following variables were entered in the proportional hazards model as possible explanatory effects to the stepwise selection algorithm: age class (18-64, 65-74, 75-79, ≥ 80), sex (M/F), PD-L1 TPS percentage indicator (< 75%, ≥ 75%), ECOG status indicator (0, > 0), antibiotics administration (yes/no), and indicators of toxicity (yes/no) for cutaneous, gastrointestinal, endocrinological, and other toxicities. Antibiotic prescriptions were only collected if they were reported in the medical records by oncologists, but were not considered if prescribed by other clinicians. Nevertheless, it was not possible to collect data on dosages, timing of administration, and whether antibiotics were administered in the weeks prior to the start of treatment and once the treatment started.

### Study measures

OS, defined in the literature as the time from the start of therapy to death or last follow-up (December 2020), was compared between patient subgroups. The number of cycles received by the patient was one of the parameters used to determine whether or not discontinuation of immunotherapy/death was related to pembrolizumab. Patients who discontinued treatment earlier than anticipated had a compromised performance status. Therefore patients were stratified according to their ECOG status (Table 1). Therefore, OS is more likely to be affected by death as a result of disease progression than by immunotherapy-related toxicity. Patients were stratified based on reported toxicity in order to provide data to assess the correlation between overall toxicity and OS. The occurrence of toxicity was investigated using clinical parameters such as blood test results, medical examinations, and symptoms reported by the patient or nursing staff (diarrhoea, nausea, rash, and/or fatigue). For the purposes of the statistical analysis, all this information was differentiated by record type and grouped into categories (gastrointestinal, endocrinological, cutaneous reactions, and others). The National Cancer Institute CTACE, version 5.0, was used to grade irAEs. To assess toxicity, we used the prescription of supporting medication (corticosteroids and/or antibiotics), the lengthened interval between treatments (> 21 days), and the grade of any type of toxicity (G1 to G3). For the purposes of this study, these and other data were recorded and retrieved analysing the administration, every 21 days.

### Statistical analysis

This observational study evaluated OS using the Kaplan-Meier method to compare patient subgroups.

A Cox proportional hazards model with stepwise variable selection was applied to identify the patient characteristics affecting OS and to estimate their hazard ratios (HR).

To perform the statistical analysis, R software was used for the Kaplan-Mayer estimation, the SAS9.4/STAT© package was used for the proportional hazard analysis, and the graphs were created using CorelDRAW software version 2018. For all statistical tests, the significance level was set to *p* < 0.05, and confidence intervals were estimated at the 95% level.

## Results

### Patient characteristics at the time of NSCLC diagnosis

The study enrolled 98 patients with mNSCLC who received pembrolizumab (200 mg q3w fixed dose) as a first-line treatment from July 2017 till February 2020. The data collected included follow-up till December 2020 to allow for a sufficient follow-up period. Patient characteristics are summarized in Table 1 below.

**Table 1:** Baseline patient characteristics

**Patients, n**	98
**Sex, n (%)**	Male, 63 (64.3); Female, 35 (35.7)
**Age, median (range)**	73 (44-89)
**Metastasis, n (%)**	
**Adrenal**	19 (19.4)
**Bone**	26 (26.5)
**Brain**	10 (10.2)
**Lymph nodes**	37 (37.8)
**Lung**	45 (45.9)
**Weight, mean (SD)**	70.06 (13.37)
**PD-L1 TPS percentage, n (%)**	
**Less than 75%**	46 (46.9%)
**Greater or equal to 75%**	52 (53.1%)
**ECOG-PS at first cycle (%)**	
**0**	25 (25.5)
**1**	64 (65.3)
**2**	9 (9.2)
**Follow-up, median in months**	13
**Duration of treatment, median in cycles**	8.5
**Overall toxicity (%)**	76 (77.5)
**Cutaneous toxicity (%)**	29 (30.1)
**Gastrointestinal toxicity (%)**	27 (27.5)
**Endocrinological toxicity (%)**	20 (20.4)
**Other toxicity (%)**	67 (68.4)

*n* Number, *SD* Standard deviation, *PD-L1* Programmed death ligand 1, *TPS* tumor proportion score, *ECOG-PS* Eastern Cooperative Oncology Group Performance Status

### Effectiveness of pembrolizumab

**Fig. 1:**
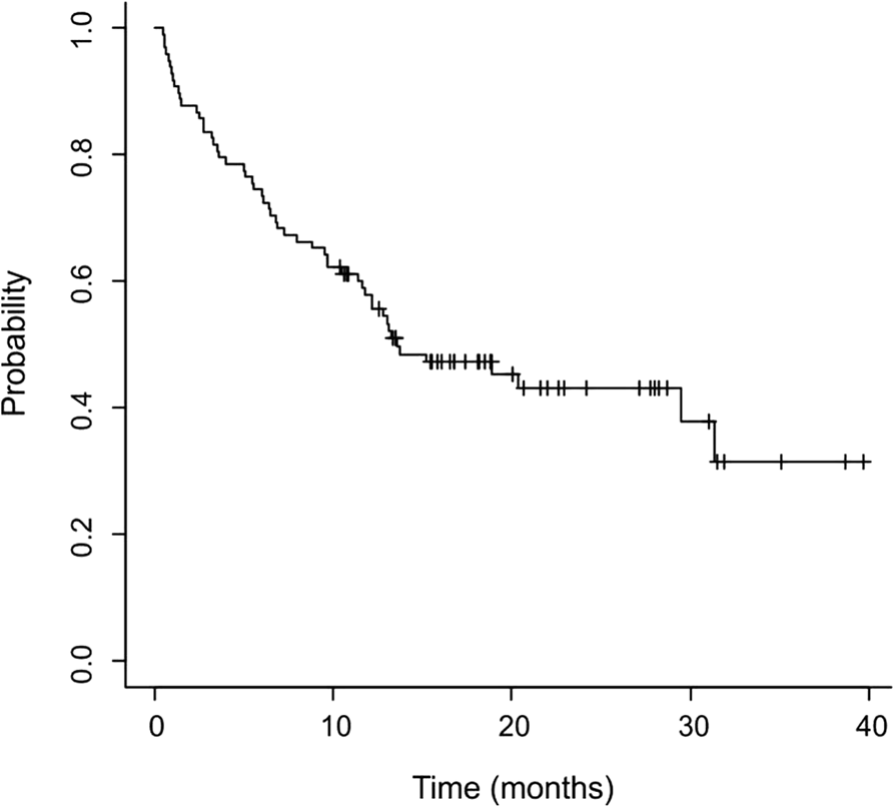
OS trend of 98 patients observed for 42 months treated with pembrolizumab. At 12 months the percentage of alive patients was 57.9% (95% IC, 48.8-68.6)

On the 31st December 2020, the median follow-up period was 13 months, the median number of cycles (each of a 3 week duration) was 8.5, and the detected median OS was 13.6 months (95% CI: 11.7-NA). As shown in Fig. 1, 57.9% of patients (95% CI: 48.8-68.6) were alive 12 months after baseline.

As discussed in the introduction, the sample size was stratified according to the most influential parameters identified in the literature.

The stepwise selection algorithm identified three variables with globally significant parameters (Wald Chi-square = 32.7, *p <* 0.0001*)*. Table 2 shows the three variables positively correlated with survival (HR < 1): ECOG status, and cutaneous and endocrinological toxicities.

**Table 2:** OS Proportional Hazards Model: significant variables and estimated parameters

Variable	Category	Degrees of freedom	Parameter estimate	SE	Chi-square (Wald)	Pr > Chi-square	Hazard Ratio (HR)	HR 95% confidence interval	
**ECOG-PS 0**	no^a^	1		*0.36*			1		
	yes		−0.810	*0*	5.070	*0.024*	0.45	*0.22*	*0.90*
**Cutaneous toxicity**	no^a^	1		*0.36*			1		
	yes		−1.019	*5*	7.783	*0.005*	0.36	*0.18*	*0.74*
**Endocrinological toxicity**	no^a^	1		*0.60*			1		
	yes		−1.712	*4*	8.040	*0.005*	0.18	*0.06*	*0.59*

*ECOG-PS* Eastern Cooperative Oncology Group Performance Status, *SE* Standard Error

^a^Reference category

**Fig. 2:**
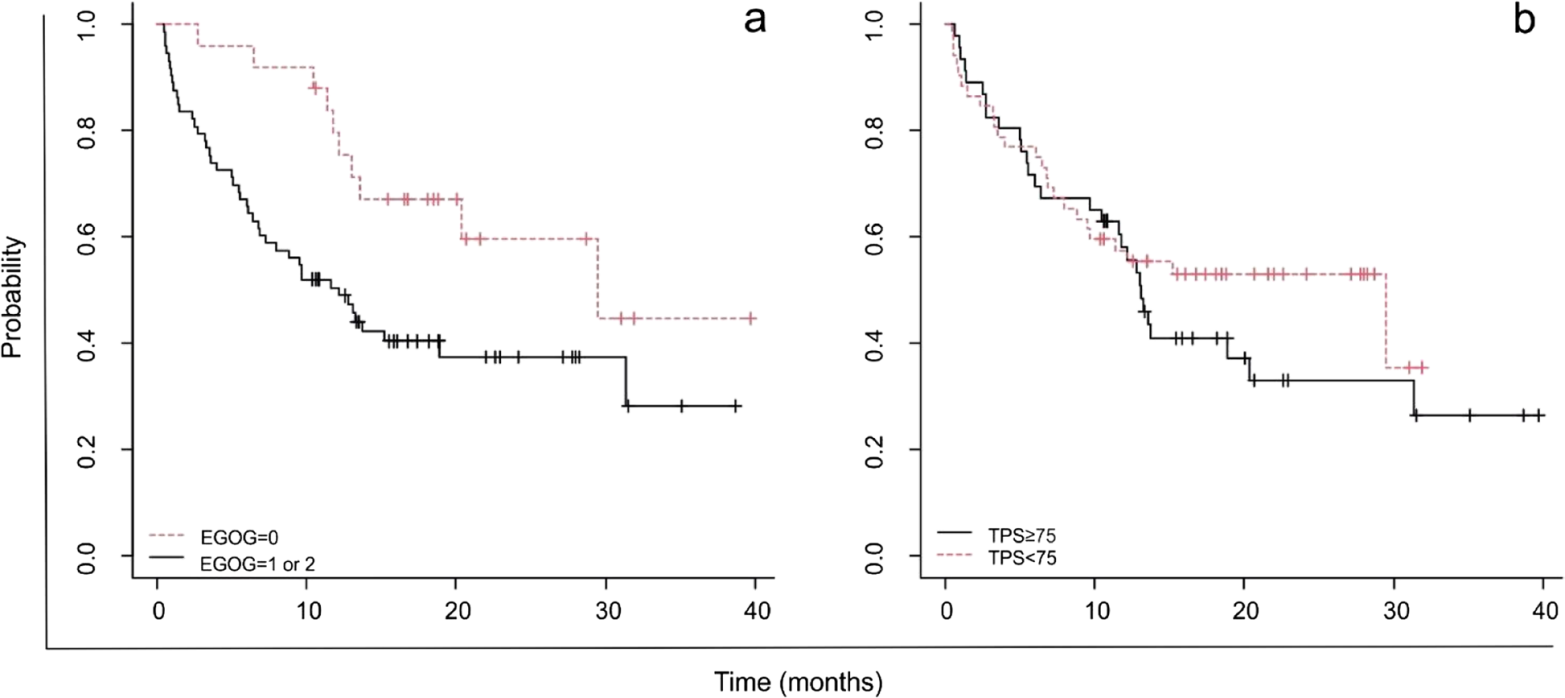
(**a**) OS analysis of 98 patients stratified by ECOG 0 (*n* =25) or 1 and 2 (*n* =73), *p* =0.02; (**b**) OS analysis of 98 patients stratified by PD-L1 TPS < 75 (*n* =46) or ≥ 75 (*n* =52), *p* =0,4

Figure 2 illustrates the OS of patients stratified by ECOG (0 v. 1 and 2) and TPS percentage (< 75 or ≥ 75). The latter is not significantly correlated to OS.

### Adverse events

During the observation period, irAEs occurred in 77.5% of patients, but no grade 4 or 5 AEs were recorded. Three (3.1%) patients discontinued pembrolizumab due to treatment-related AEs. The following percentages of AEs were recorded in this study: 30.1% cutaneous, 27.5% gastrointestinal, and 20.4% endocrinological.

**Fig. 3:**
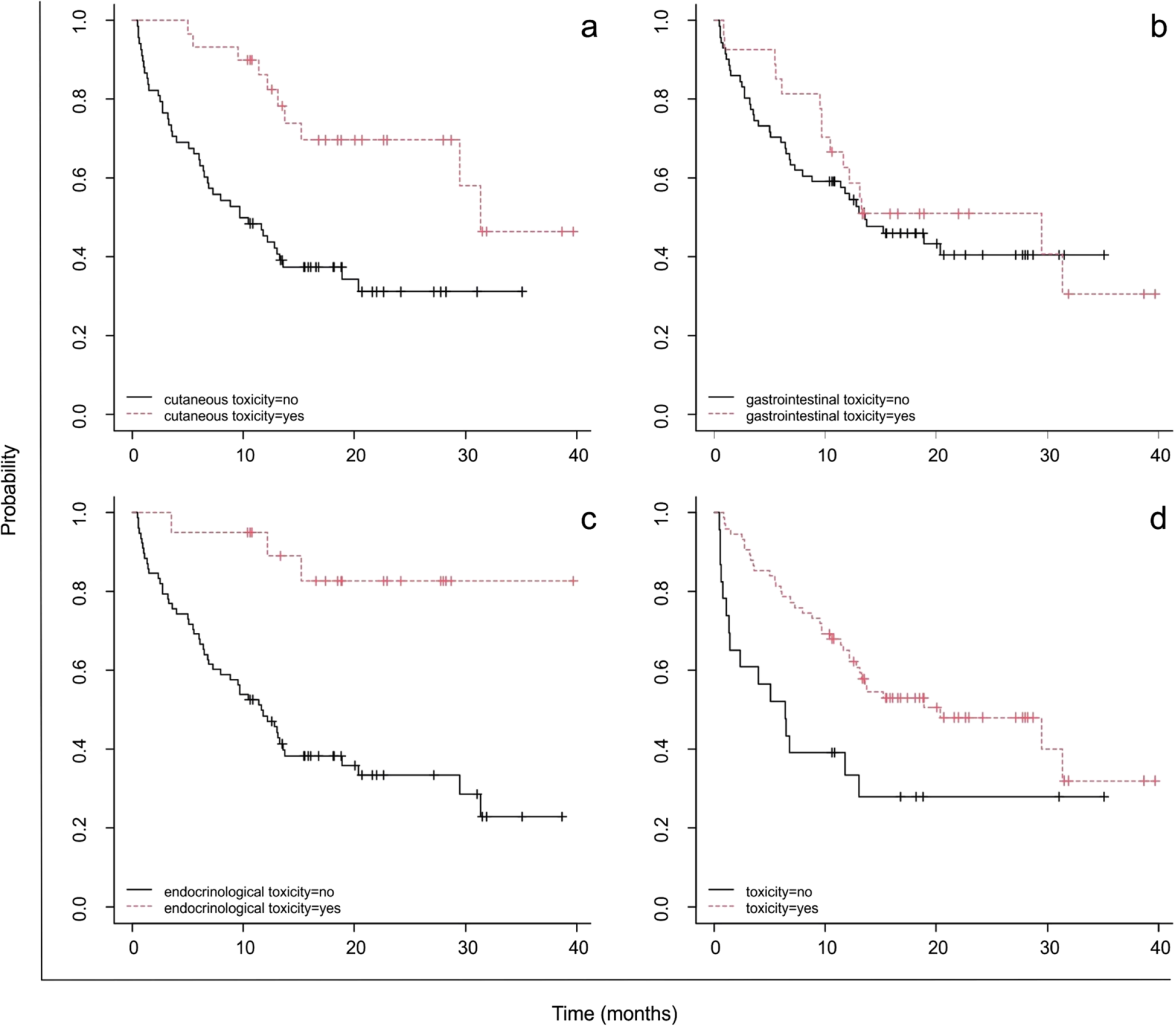
OS trend in (**a**) patients with (*n* =30) or without (*n* =68) treatment-related cutaneous toxicity (*p* =0.0008); (**b**) patients with (*n* =27) or without (*n* =71) treatment-related gastrointestinal toxicity (*p* =0.5); (**c**) patients with (*n* =20) or without (*n* =78) treatment-related endocrinological toxicity (*p* =0.0005); and (**d**) patients with toxicity (*n* =75) or without toxicity (*n* =23) (*p* =0.006)

Patients with overall toxicity showed a significantly higher median OS (20.39 months, 95% CI: 13.08-NA) than those with no toxicity (6.46 months, 95% CI: 1.41-NA, *p* = 0.006), as described in Fig. 3d. Moreover, patients without cutaneous toxicity reported a reduced OS (10.1 months, 95% CI: 6.52-18.9) compared to those with (31.3 months, 95% CI: 29.44-NA, (*p* = 0.0008), and both patients without endocrinological toxicity showed a significantly reduced OS (11.7 months, 95% CI: 8.0-18.9, *p* = 0.0005).

**Fig. 4:**
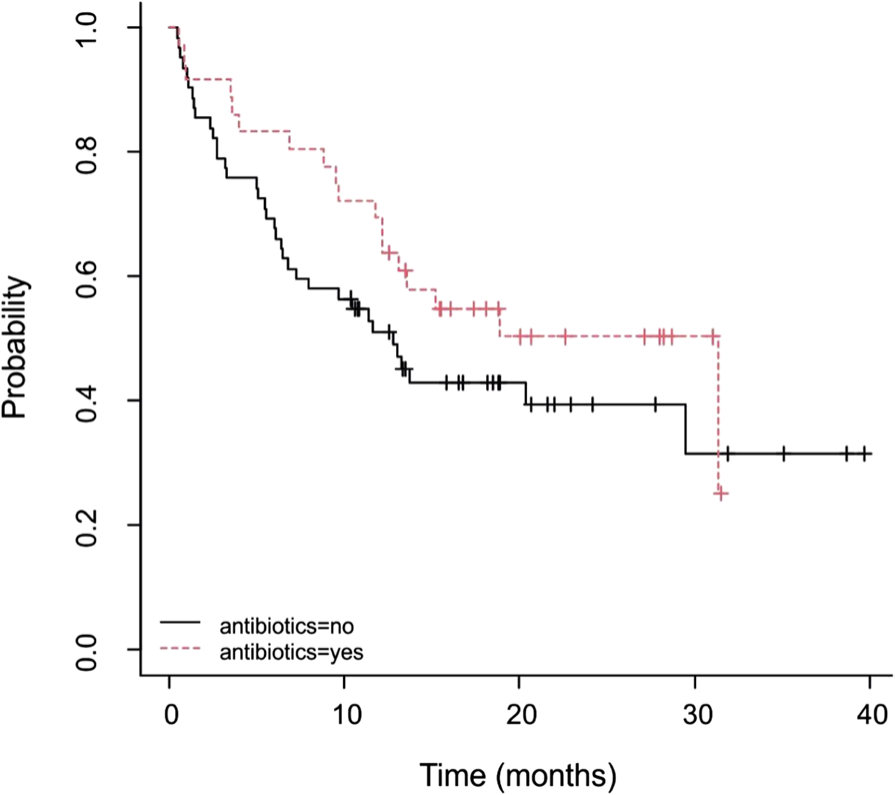
OS in patients treated with antibiotics (36.7%) during pembrolizumab therapy (*p* =0.2)

Furthermore, as regards the impact of concomitant therapies, the association between antibiotic administration and OS have been evaluated. As shown in Fig. 4, the OS in patients who were not administered antibiotics was 31.3 months (95% CI: 12.20-NA), while the OS in patients who were administered antibiotics was more than halved (12.8 months, 95% CI, 7.31-NA). In opposition, the use of corticosteroids had no impact on OS (*p* > 0.05).

The data obtained from the literature concerning the toxicity profile in two clinical trials were compared with the data retrieved from this study. As shown in Table 3, the real-world study confirmed the toxicity of pembrolizumab, indicating a higher incidence of cutaneous and endocrinological events compared to the two registered clinical trials.

**Table 3:** Summary of the literature review on the toxicity profile of pembrolizumab

	Keynote-042 [9] %	Keynote-024 [8] %	Real-world study %
**Cutaneous Toxicities**	14.0	22.1	30.1 (*p* = 0.0008)
**Endocrinological Toxicities**	17.0	17.5	20.4 (*p* = 0.0005)
**Gastrointestinal Toxicities**	13	32	27.5 (*p* = 0.5)
**Overall Toxicities**	63	76.6	77.5 (*p* = 0.006)

As a result of the proper management of AEs in routine clinical practice, no patient died from an adverse event when compared to 1 patient in KEYNOTE-024 and 13 patients (2%) in KEYNOTE-042.

## Discussion

This real-world retrospective observational study assessed pembrolizumab as a first-line treatment option for mNSCLC among patients with genomics characteristics of TPS ≥50% and no sensitizing EGFR or ALK translocations.

Results showed a median OS of 13.6 months, which corresponded to the median OS estimated in another real-world trial with OS of 15.2 months [12]. This paper’s additional value also consists in a longer follow-up period (42 months).

Apart from the clinical benefits, several studies support the direct relationship between the activity of ICIs and the onset of immune-related side effects. The latter are distinct from the side effects commonly associated with traditional chemotherapy, such as nausea, vomiting, mucositis, or alopecia. The onset of irAEs may request delayed administration, or even treatment discontinuation. To avoid jeopardizing treatment outcomes, it is critical to detect and manage those manifestations promptly. In order of frequency, the most common locations of irAEs are cutaneous and gastrointestinal, but also endocrinological, pulmonary, cardiac, and neurological [13, 14]. These events require close monitoring. Therefore, patients and caregivers must be educated to immediately report the onset of an outbreak. As previously mentioned, early detection and treatment can have a reversing effect on the outcomes. However, as highlighted in this study, irAEs are detected at a late stage in a real-world setting, ultimately resulting in the administration of corticosteroids. In fact, out of the 98 patients enrolled in the study, 62.2% were treated with a loading dose of corticosteroids (prednisone and dexamethasone) followed by tapered doses. Corticosteroids and prognosis in patients treated with ICIs are an exclusion parameter that may preclude initiating therapy in the early phases. According to the literature, the threshold dose of prednisone is 10 mg/day; the relationship is so strong that clinical trials administering pembrolizumab do not allow the concomitant use of prednisone, given that it can impair the medication’s activity and outcomes. It is also important to emphasize that the rationale for the use of corticosteroids prescribed to treat cancer-related (oedema and fatigue) or treatment-related (immunity over response) conditions is quite different. As described by Ricciuti et al., the use of prednisone (> 10 mg/day) does influence OS after 5 years (OS of 4.5 months for the group treated with corticosteroids versus 11.5 months for the non-treated group *p* < 0.001). These data were retrieved from a cohort treated with corticosteroid at baseline for pre-existing comorbidities or for the treatment of cancer-related conditions. In the above-mentioned study, patients were stratified based on the palliative or non-palliative use of prednisone. In the first case, OS was significantly reduced, but the consequent stratification showed that no significant influence was found on the outcomes in the second group. These findings emphasize that the use of prednisone at baseline is not linked to the effectiveness of pembrolizumab. However, the palliative setting frequently results in a poor prognosis, regardless of prednisone use. Hence, it is not confirmed that prednisone should be considered as an exclusion criterion in the eligibility assessment of patients with mNSCLC with PD-L1 expression [15]. In our analysis, patients received corticosteroids in a non-palliative setting, with no correlation on the OS (*p* > 0.05).

In this environment, the role of clinical pharmacists [16] becomes critical for the recognition and management of irAEs, given their involvement in the treatment decision-making process. Nonetheless, different studies underlined the importance of a multidisciplinary approach to promptly detect AEs of different grade. The inclusion of different healthcare professionals paves the way to a wide-ranging view on the patient’s symptoms and, in this way, determine with greater certainty which symptoms may be related to an adverse event and which are related to the underlying pathology [17]. Hence, in the oncological setting the active presence of the clinical pharmacist should be implemented and set at bedside. Through the close relation with the patient and the devoted time to him, the clinical pharmacist can recognize uprising AEs or, potentially, prevent them. Additionally, recent findings have highlighted that the development of irAEs is a strong predictor of survival outcomes in NSCLC. Thus, patients who experienced irAEs had improved response rates in terms of OS [18], as was also confirmed in this real-world study. In particular, Gulati et al. [19] demonstrated that cutaneous toxicity is significantly associated with improved OS (*p* < 0.001). This result is confirmed by this study’s findings, which revealed a statistically significant *p-value* of 0.0008. Thus, correlation between improved OS outcome and cutaneous (RR 0.361, 95% CI: 0.176-0.738) and endocrinological toxicity (RR 0.180, 95% CI: 0.055-0.589) was further confirmed. Among patients enrolled in the study no additional examinations were carried out on cutaneous lesions, likely because the nature of the lesion itself did not suggest the need of further investigation.

The comparison with data from recent literature revealed a high degree of similarity. This study detected a rate of any grade of AEs (77.5%), comparable to the percentage reported in KEYNOTE-024 and KEYNOTE-042 (76.6 and 63%, respectively). However, few differences in the incidence of specific irAEs were noted between clinical trials and this real-world study. In particular, 14% of patients in KEYNOTE-042 experienced cutaneous reactions (rash and pruritus) and 22.1% in KEYNOTE-024, whereas 30.1% of patients in this real-world setting were affected by all types of cutaneous events. An overall similar result was obtained with regard to endocrinological toxicity (17% in KEYNOTE-042, 17.5% in KEYNOTE-024 v. 20.4% in this study). In opposition to the clinical trial setting, real life analysis involves people that are not required to follow diet or test protocols that may influence the incidence of cutaneous reactions. Gastrointestinal events (diarrhoea, nausea, vomiting, and constipation) were more common in the present study group if compared with KEYNOTE-042 (27.5% v. 13%) but similar to KEYNOTE-024 (32.4%).

Similarly, the rate of treatment discontinuation due to AEs was 3.1% in this analysis (similar to the 3.7% reported in the Pembreizh study [12] v. 7.1 and 9% in Randomised Clinical Trials [8, 9]). On this point, it should be noted that, while the clinical practice environment enabled us to analyse what really happened to patients, it does not require the close follow-ups and scheduled monitoring visits that are necessary in the trials. Therefore, data collected in the real-world setting can be biased and the frequency of irAEs may be underestimated.

Antibiotics play a key role in the treatment of immune disorders and unrelated infections in the management of irAEs. Concomitant exposure to broad-spectrum antibiotic therapy may adversely influence clinical outcomes [20] due to the alteration of intestinal microbiota [21]. The alteration of intestinal microbiota, described as a key host determinant of response to ICIs, is known as the precondition for cancer-specific immunity [22].

This analysis also evaluated the association between antibiotic consumption and overall survival. Recent studies [23, 24] investigated the relationship between the influence of antibiotic use and microbiota response on oncological treatments but could not prove any significant evidence. According to this study, the administration of antibiotics appeared to benefit patients, suggesting a higher OS in patients treated with these medications, but results were not significant (Fig. 4). The direct influence of altered microbiota and treatment outcomes is yet to be confirmed by further studies. As regards the management of toxicity related to the treatment no specific therapies in addition to corticosteroids and antibiotics were detected.

Apart from the absence of EGFR and ALK mutations, the possibility of first-line treatment with pembrolizumab is contingent upon a minimum 50% PD-L1 mutation as per indication requirements. It is for this reason that this parameter is frequently used as a stratification indicator. Indeed, the PD-L1 level was used as a discriminator paradigm in the recent systematic review conducted by Kim et al [25] and in Aguilar et al. study [26].

It is interesting to note that the present real-world study discovered a link between improved OS and the ECOG status, which describes the patient’s overall comprehensive clinical condition (Fig 2a, *p =* 0.02). This was also confirmed by the Chi-square test, which showed a positive correlation (RR 0.445, 95% CI: 0.220-0.900).

As expected, this real-world observational study has some limitations. These include the sample size, which was exclusively made up of Caucasian patients, the outcomes, and the fact that irAEs were reported by oncologists and nurses only. As a result, it is impossible to rule out that significant data may have been lost and that errors and omissions may have occurred.

Moreover, not all the irAEs were strictly graded according to CTCAE 5.0, and omissions may have resulted in slightly biased results. All these limitations may have precluded other interesting findings or have reduced the spectrum of adverse reactions, however it reflects the reality of the IOV-IRCCS, as regards the medical and therapeutic management.

Finally, a larger sample size and a more comparable follow-up period would have improved the analysis. This monocentric study could be expanded to a multicentre real-world study, incorporating additional clinical landmark centres in order to collect more data, resulting in a larger collection of clinically and statistically significant data.

## Conclusions

These findings corroborate the correlation between OS and toxicities occurring in patients treated with pembrolizumab in a real-world setting. The percentage of irAEs was comparable to the percentages reported in KEYNOTE-024 and KEYNOTE-042. This indicates that the immune system activation caused by ICIs is likely to manifest in the form of cutaneous reactions that improve OS. The percentage of patients reporting cutaneous reactions was higher in this real-world study than in clinical trials.

Furthermore, data confirmed that there is no correlation between TPS percentage and OS, as reported in the results section.

## Data Availability

The data underpinning the findings of this work are available from the corresponding author on reasonable request.

## Abbreviations

AEs: Adverse Events
ALK: Anaplastic Lymphoma Kinase
CI: Confidence Interval
CTCAE: Common Terminology Criteria for Adverse Events
GCP: Good Clinical Practice
ECOG-PS: Eastern Cooperative Oncology Group - Performance Status
EGFR: Epidermal Growth Factor Receptor
HR: Hazard Ratio
ICIs: Immune Checkpoint Inhibitors
IRCCS: Scientific Institute for Research
IOV: Veneto Institute of Oncology
irAEs: Immune-Related Adverse Events
mNSCLC: metastatic Non–Small Cell Lung Cancer
NA: Not Available
NSCLC: Non–Small Cell Lung Cancer
OS: Overall Survival
PD-1: Programmed Death-1
PD-L1: Programmed Death-Ligand 1
RCT: Randomised Clinical Trials
RR: Relative Risk
SD: Standard Deviation
SE: Standard Error
TPS: Tumour Proportion Score
WHO: World Health Organization
